# Antibacterial *Aloe vera* Based Biocompatible Hydrogel for Use in Dermatological Applications

**DOI:** 10.3390/ijms24043893

**Published:** 2023-02-15

**Authors:** Mariana Chelu, Adina Magdalena Musuc, Ludmila Aricov, Emma Adriana Ozon, Andreea Iosageanu, Laura M. Stefan, Ana-Maria Prelipcean, Monica Popa, Jose Calderon Moreno

**Affiliations:** 1 “Ilie Murgulescu” Institute of Physical Chemistry, 202 Spl. Independentei, 060021 Bucharest, Romania; 2 Department of Pharmaceutical Technology and Biopharmacy, Faculty of Pharmacy, Carol Davila University of Medicine and Pharmacy, 6 Traian Vuia Street, 020945 Bucharest, Romania; 3 Department of Cellular and Molecular Biology, National Institute of R&D for Biological Sciences, 296 Splaiul Independentei, 060031 Bucharest, Romania

**Keywords:** *Aloe vera*, wound healing, hydrogels, green synthesis, allantoin, xanthan gum, antimicrobial action

## Abstract

The present research aims to describe a new methodology to obtain biocompatible hydrogels based on *Aloe vera* used for wound healing applications. The properties of two hydrogels (differing in *Aloe vera* concentration, AV5 and AV10) prepared by an all-green synthesis method from raw, natural, renewable and bioavailable materials such as salicylic acid, allantoin and xanthan gum were investigated. The morphology of the *Aloe vera* based hydrogel biomaterials was studied by SEM analysis. The rheological properties of the hydrogels, as well as their cell viability, biocompatibility and cytotoxicity, were determined. The antibacterial activity of *Aloe vera* based hydrogels was evaluated both on Gram-positive, *Staphylococcus aureus* and on Gram-negative, *Pseudomonas aeruginosa* strains. The obtained novel green *Aloe vera* based hydrogels showed good antibacterial properties. In vitro scratch assay demonstrated the capacity of both AV5 and AV10 hydrogels to accelerate cell proliferation and migration and induce closure of a wounded area. A corroboration of all morphological, rheological, cytocompatibility and cell viability results indicates that this *Aloe vera* based hydrogel may be suitable for wound healing applications.

## 1. Introduction

The global evolution of the pandemic and climate changes have led to an increase in consumer health awareness worldwide [1,2]. Currently, an important research direction worldwide is to develop novel strategies to promote and design newer environmentally friendly materials [3,4,5]. The goal is to be produced on a large scale at low cost, with low hazardous waste generation and highly functional remedial activity [6,7,8]. In the last decade, in the treatment of skin diseases, the demand for products with plant-based or organic polysaccharide ingredients has increased [9,10]. Choosing the perfect wound dressing is a crucial step that ensures the speed of regeneration of the skin wound as well as the avoidance of microbial contamination [11,12,13]. In dermatology, studies on functional skin wound care products and skin diseases are of great interest, with a focus on wound dressings (dry and wet products). There is a permanent interest in obtaining new and effective materials for skin wound healing and tissue regeneration using natural raw materials with effective biological activity in order to improve the quality of life.

The use of hydrogels, a 3D cross-linked hydrophilic polymeric network, as different types of wound dressing materials is due to their excellent biocompatible properties as well as their ability to absorb and retain hundreds of times their weight in water by maintaining their structure [14,15]. Accordingly, a perfect wound dressing is deemed to be one that meets the following requirements such as biodegradability, biocompatibility, antimicrobial, super absorbency and, last but not least, the avoidance of scarring [16,17,18,19].

Both synthetic and natural biopolymer-based materials have been the most widely used bioresources for manufacturing effective wound care systems due to their biocompatibility, biodegradability, solubility and ease of fabrication, immobilization of bioactive compounds, cost-effectiveness and characteristic hydrophilicity in conjunction with a wide range of organic functionalities [20,21].

Allantoin is a naturally occurring chemical compound contained in many plants, such as bearberry, horse chestnut and comfrey [22]. Among its many skin care benefits can be mentioned the reduction of skin irritation, anti-inflammatory properties and wound healing properties [23,24]. Numerous clinical studies have shown that allantoin has both a moisturizing effect and a strong stimulation of cell proliferation and tissue regeneration [25,26]. The beneficial characteristics of this bioactive compound justify its utility and applications in numerous compositions formulated for the cosmetic industry, for the treatment of chronic wounds such as ulcers or for the healing of injuries, wounds and burns [27,28,29,30].

Xanthan gum, a water-soluble product obtained by fermentation of Gram-negative bacterium named *Xanthomonas campestris*, is characterized by a high molecular weight with values in the range of 2 × 10^6^ and 20 × 10^6^ Da [31]. Due to its internal structure based on 3D association of molecular chains, aqueous solution of xanthan gum presents weak properties characteristic to gels [32]. Its application in the food industry is reported from 1969 due to its non-sensitizing, non-toxic, and emulsion stabilizer properties. In recent years, xanthan gum has been used extensively in the biomedical field due to its biodegradable, gelling and biocompatible properties [33].

*Aloe vera*, a medicinal plant that belongs to the Liliaceae family, is widely used in cosmetics, food products and medicine [34]. The global *Aloe vera* hydrogel market was assessed at USD 337.7 million in 2022 and is expected to reach USD 712.3 million by 2032, at a compound annual growth rate (CAGR) of 7.8% from 2022 to 2032, higher than the estimated CAGR for the period 2017–2022 (of approximately 6%) [35]. Due to its composition, water (>98%) in the mucilaginous gel and ~2% minerals, vitamins, enzymes and polysaccharides (such as hemicellulose, cellulose, mannose derivatives, pectin, glucomannan and acemannan), *Aloe vera* possesses numerous antibacterial, antioxidant, antiviral, antiallergic, UV protection, cicatrizing, anti-inflammatory and antiviral therapeutic properties [36,37,38,39,40].

Over the years, various types of hydrogel-based biomaterials containing xanthan gum, allantoin or *Aloe vera* mixed with synthetic polymers to be used in the healing process of wounds were reported. Oh et al. reported a hydrogel-containing, oxidized alginate and gelation into which was incorporated chitoologosaccharide and salicylic acid for wound healing applications [41]. It was demonstrated that the salicylic acid which was grafted in the chitoologosaccharide backbone has the capacity to increase the hydrogel antioxidant activity. The in vivo experiments on a mouse wound model have shown no cytotoxic effect and an acceleration of the wound healing process. From the literature, it is known that salicylic acid has anti-inflammatory activity and bioavailability [42,43] and can accelerate the wound healing process in rats [44].

Pereira et al. have developed thin hydrogel films based on alginate combined in different proportions with *Aloe vera* gel (95:5, 85:15 and 75:25, *v*/*v*) and using calcium ions (5% solution of calcium chloride) as cross-linking agents [45] for wound healing applications. The physical–chemical characterization of prepared hydrogel films have shown adequate mechanical properties and an insolubility after 24 h in water. The presence of *Aloe vera* conducted an improvement in the transparency and a good thermal stability. In higher concentration, *Aloe vera* leads to an improvement in the swelling behavior and water absorption. Salehi et al. [46] developed a hydrogel-based chitosan (2% *w*/*v*) which contains *Aloe vera* gel (0.5% *v*/*v*) and ethylenediaminetetraacetic acid (EDTA) (0.01% *w*/*w*) by cross-linked reaction (1 chitosan and 6 β-glycerol phosphate, β-GP *w*/*w*) for use as wound-healing material. In vivo wound healing efficacity using an animal wound model demonstrated both an acceleration of the healing process and the antibacterial and proliferative properties of *Aloe vera* gel. Different synthetic hydrogels have also been synthesized and studied for the wound care system. In one study, nanofibers were fabricated using poly-vinylpyrrolidone (PVP) and *Aloe vera* or *Aloe vera* acetate in order to determine the efficiency of skin tissue regeneration and their use as biocomposite nanofibrous scaffolds [47]. In a recent study, new antimicrobial membranes, composed of hydrogels based on a synthetic polymer, poly (vinyl alcohol) and *Aloe vera*, incorporating curcumin and gentamicin, were obtained through a new physical crosslinking method. The antibacterial activity was tested, and the wound healing and skin regeneration capacity of these membranes was shown [48].

Allantoin was used in hydrogel films of chitosan and polyvinyl alcohol cross-linked by the freeze-thaw method. These films, which also contain honey, were prepared for the repair of skin wounds. The physicochemical properties, the long-term biocompatibility, the release of allantoin from the hydrogel as well as the antimicrobial activity of the films were investigated. Following the study, the obtained results suggested that the prepared materials can be applied for wound healing and skin tissue engineering [49]. Recently, new scaffolds based on chitosan/gelatin containing different percentages of allantoin were made to be used in the wound healing process, including severe wounds or burns. The prepared materials showed antibacterial activity against *S. aureus* and *E. coli* bacteria and indicated appropriate scaffold properties for cell adhesion [50]. 

Xanthan gum and polyacrylamide were used to develop hydrogel for the use as wound dressings by one-pot method [51]. The authors have demonstrated the water uptake efficiency of the polyacrylamide-xanthan gum hydrogels in addition to their self-healing ability, universal adhesion, biocompatibility and use as a platform for bioactive molecule delivery. A thermo-reversible hydrogel based on different ratios (50/50 and 60/40) between xanthan gum and konjac glucomannan (at different concentrations (1% and 2% *w*/*v*) was manufactured and characterized. The results highlighted good physical, chemical and biological properties being suitable for their future use in wound dressings fabrication [52]. Pagano et al. [53] has developed and characterized a bioadhesive film prepared by the casting method, using xanthan gum (1% *wt/wt*) and sodium alginate (1.5% *wt/wt*). The two compounds were used in a 2.5/7.5 (*wt/wt*) ratio. As a plasticizing agent, glycerol (10% *wt/wt*) was used. The obtained results revealed a good capacity to absorb a simulated wound fluid (~65% *wt/wt* within 1 h). The obtained hydrogel can be applied in wound treatment for exuding wounds.

Skin injuries such as burns are potentially fatal and a physically debilitating trauma, leading to chronic disability. One of the main causes of delayed wound healing is microbial infections. *Staphylococcus aureus* is a major human pathogen that causes severe infections, especially in immunocompromised and long-term hospitalized patients [54]. *Pseudomonas aeruginosa* is an opportunistic organism that is also increasingly recognized as an important multidrug-resistant nosocomial pathogen [55]. To meet this challenge, the potential use of allantoin, *Aloe vera*, salicylic acid with the role of keratolytic agent, and xanthan gum, used as a gelling agent, was investigated for biomedical application for the treatment of skin wounds.

The novelty of this research is represented by the manufacturing of new formulations based on natural and bioavailable components, unlike previous literature studies when the hydrogels were prepared by mixing natural and synthetic materials. The use of bioactive natural polymers can avoid side effects associated with synthetic polymers. Consequently, the main purpose of the present article was to design and to prepare new functional green materials, such as hydrogels, that have multiple therapeutic benefits to be used in the biomedical field. To the best of our knowledge, this research is the first report regarding the manufacturing of *Aloe vera* based hydrogel containing allantoin, xanthan gum and salicylic acid as a new wound healing system. Thus, two biodegradable natural hydrogels were prepared by an all-green solution method based on natural ingredients and employing two concentrations of *Aloe vera* as a bioactive compound. The bioactive compounds play an important role in the healing process of scratch wounds and could lead to the adjustment of inflammation, to the intensification of the epithelization of the wound and, finally, to the regeneration of the tissue.

## 2. Results and Discussion

### 2.1. Organoleptic Properties

The organoleptic characteristics of the AV5 and AV10 hydrogels were visually analyzed with regard to appearance and texture. AV5 has a light cream color and AV10 has a medium cream color.

**Figure 1:**
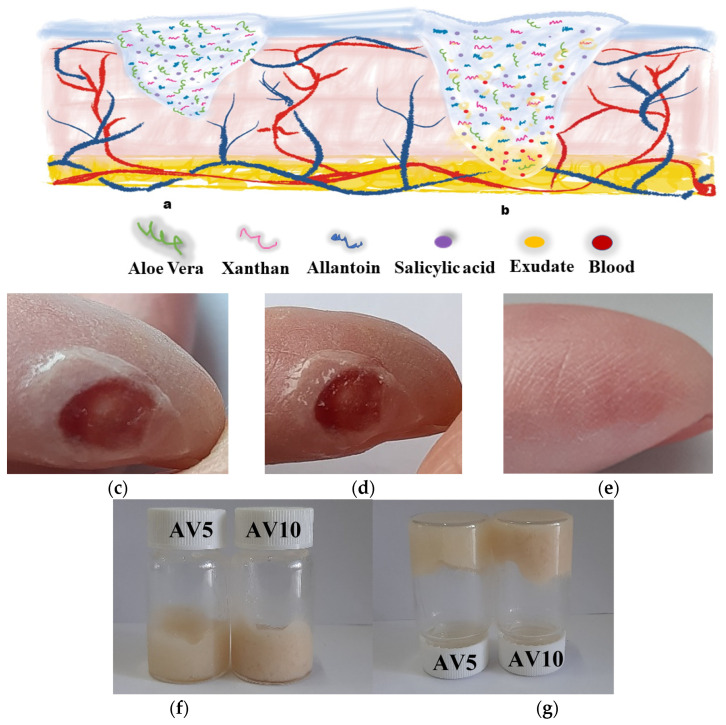
Schematic illustration of *Aloe vera*-based hydrogel and the potential use as a wound dressing: (**a**) dry and (**b**) wet; Images of a wound during the healing process using *Aloe vera* hydrogel: (**c**) initial time; (**d**) after 5 min; (**e**) the wound was healed after 20 days; (**f**,**g**) Photographs showing the formation of the hydrogel’s measurement by inverted vial method.

The obtained hydrogel based on *Aloe vera* can be applied to a skin wound in both dry and wet state (Figure 1a,b). Figure 1c–e represent the images of the wound during the healing process at different healing times. It was observed that after 5 min the *Aloe vera* based hydrogel completely adhered and interacted with the wound and the surrounding tissue (Figure 1d). The wound was completely healed (Figure 1e) 20 days later.

The hydrogels formation which possessed a rheological structure was immediately briefly evaluated by the test vial inversion method [56,57,58] by macroscopic analysis of the properties of not flowing and not deforming under its own weight (Figure 1f,g). The two developed hydrogels were viscous, homogeneous and presented no deformation or flow when the vial was inverted.

### 2.2. pH Analysis

In terms of pH values AV5 and AV10, wet and dry hydrogels, proved to have approximatively the same value (Table 1). The obtained values demonstrate that the AV concentration didn’t influence the pH of the obtained hydrogels. The studied samples proved to have a slight acid nature, the same as the pH of the skin, which ranges from 4.1 to 5.9 being dependent by gender or body part, demonstrating that the products will be well-accepted and will not produce irritations [59].

### 2.3. Rheological Analysis

**Figure 2:**
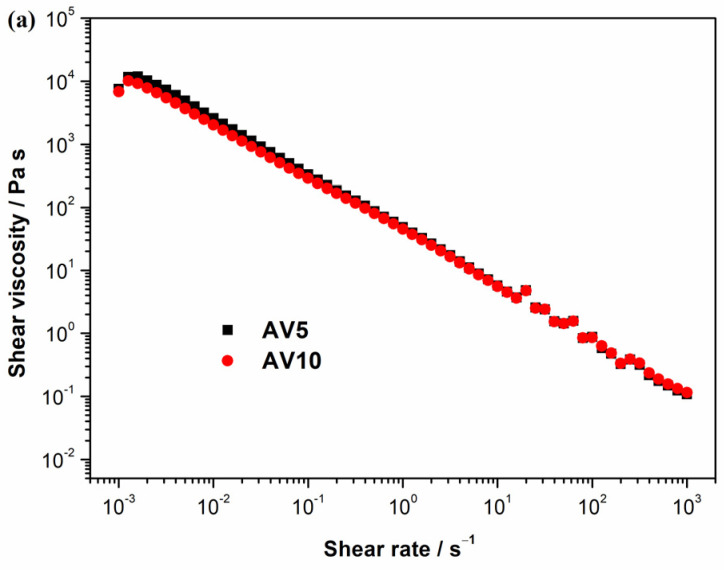

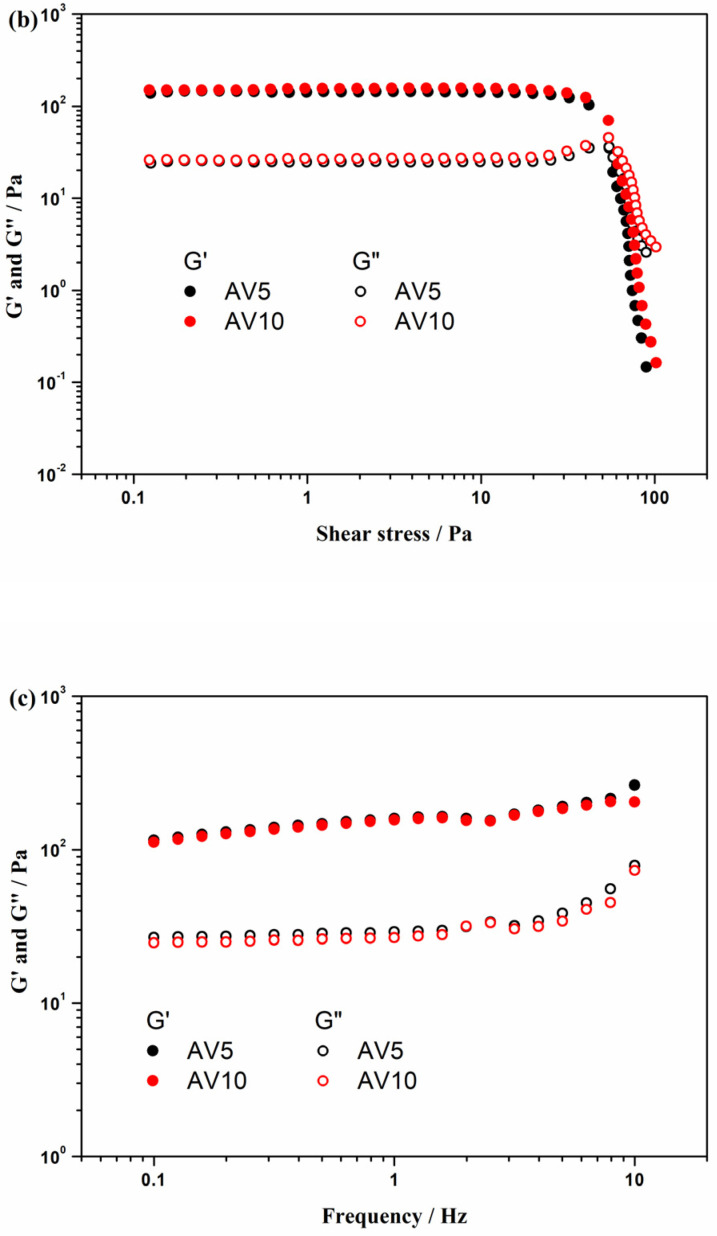
(**a**) Shear viscosity vs. shear rate of the AV5 and AV10 samples; (**b**) Storage (G’) and loss (G”) moduli as a function of shear stress at 1 Hz for AV5 and AV10 samples; (**c**) Storage (G’) and loss (G”) moduli as a function of frequency at 5 Pa for AV5 and AV10 samples.

The shear flow resistance of AV5 and AV10 materials was tested, and the results are shown in Figure 2a as viscosity vs. shear rate.

As the shear rate increased, the viscosity of both materials decreased greatly, implying that the higher the shear rate, the lower the viscosity. This result indicates the presence of a non-Newtonian shear thinning character throughout the 10^−3^ to 10^3^ s^−1^ domains. Likewise, the difference in viscosity between samples AV5 and AV10 is not significant. To identify the linear viscoelastic region, amplitude sweep experiments are required. The results demonstrate a pseudoplastic rheological behavior for the two *Aloe vera* based hydrogels. This demonstrates that when a shear stress is applied, the hydrogel becomes fluid, and, in this way, the spreading is facilitated. Similar results were obtained by Jales et al. [60] and Medina-Torres et al. [61]. Jales et al. developed *Aloe vera* mucilaginous-based hydrogels, which contain 1% Carbopol or 2% hydroxyethylcellulose, as the gelling agent, for use in the treatment of psoriasis. Medina–Torres et al. also obtained a shear-thinning non-Newtonian behavior for the solutions of *Aloe vera* mucilaginous freeze-dried gel with xanthan gum. Figure 2b shows the results of determining the linear viscoelastic region (LVER) using amplitude measurements at different shear stresses and a constant frequency of 1 Hz. The constant plateau of G’ and G” indicates the linear viscoelastic region for AV5 and AV10, which is in between 0.1 and 20 Pa and is followed by a decrease in G’. As a result, the relationship between shear stress and G’ is no longer linear, and the samples become soft. Since G’ is bigger than G”, the samples have solid or gel-like rheological profiles. Additionally, we can notice that beyond a certain shear stress (around 50 Pa), G’ intersects with G”, indicating that the samples begin to flow (the yield point). Figure 2c lists the storage (G’—elastic part) and loss (G”—viscos part) moduli responses as a function of frequency at 5 Pa for samples AV5 and AV10. Similar viscoelastic properties of the *Aloe vera* gel obtained from leaf as purified pulp, which is further used to produce Carrisyn hydrogel wound dressing, are reported [62].

Frequency sweep test results indicate a more elastic than viscous behavior (G’ > G”), and because both rheological moduli are not frequency dependent, especially at low frequencies, samples AV5 and AV10 present a similar rheological profile where the elastic gel character predominates. The same results regarding the elastic behavior are also reported by Medina–Torres et al. for the *Aloe vera* mucilaginous with xanthan gum. Rajinder observed an increase in elasticity for concentrations of xanthan group above 0.03 g/m [63].

### 2.4. SEM Analysis

**Figure 3:**
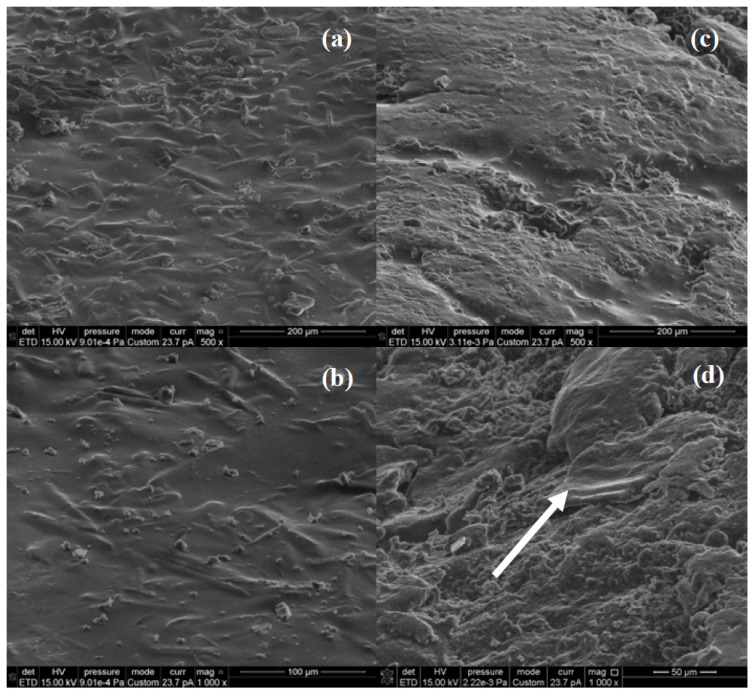
SEM images of AV5 (**a**,**b**) and AV10 (**c**,**d**) samples.

The morphology of the dried AV5 and AV10 hydrogels is that of homogeneous solids (Figure 3). Although both materials have relatively smooth surfaces [64], AV5 exhibits typical fiber-like microstructures that appear embedded in the gel, aligned in the plane of the surface (Figure 3a,b), and AV10 SEM images (Figure 3c,d) also show plate-like morphologies on the surface, such as the one indicated by an arrow in Figure 3d.

### 2.5. Antimicrobial Activity of Hydrogels Based on Aloe vera

The two hydrogel samples did interfere with microbial growth. *S. aureus* proved to be more susceptible, with inhibition diameter up to 9 mm (Table 2). Similar results were obtained against clinical isolates *S. aureus*, *S. epidermidis*, and *Pseudomonas aeruginosa* when testing aloe root and leaf extracts [65]. Among the two samples, AV10 seems to be more efficient against microbial growth.

**Figure 4:**
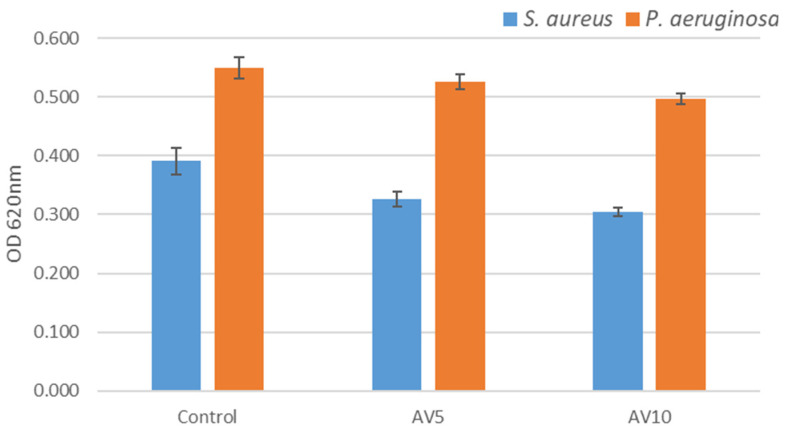
The effect of the two types of hydrogels on the bacterial growth at 24 h. Results are shown as mean ± deviation standard, n = 3.

In terms of microbial growth monitoring, OD620 nm (the microbial growth could be monitored by measuring the optical density of the culture at the wavelength of 620 nm, at 24 h of cultivation) signalled the antimicrobial potential for both hydrogel variants and against both strains. Nevertheless, the trend was maintained, and *S. aureus* was considerably more sensitive to the aloe based hydrogels when compared to the untreated control (Figure 4). The mechanism of action of the aloe based hydrogels’ antibacterial activity can be linked to the disruption in the microbial membrane in contact with the polysaccharides-based gels, as previously reported by Filip et al. [66]. The synergy of *Aloe vera*, allantoin and xanthan gives the considerable antibacterial effect against the Gram-positive *S. aureus.*

It is known that *Aloe vera* has potent antibacterial, antifungal and antiviral properties along with the anti-inflammatory and wound healing capacity. The antimicrobial effect has been attributed to the anthraquinones content [67]. The AV-based hydrogels exhibit biomedical potential for topical applications mainly due to their regenerative and antimicrobial properties. For instance, hybrid nanofibers loaded with AV plant extract developed for wound care management displayed in vivo wound-healing potential and possessed antimicrobial properties against *Staphylococcus aureus* (*S. aureus*) and *Escherichia coli* (*E. coli*) [68].

### 2.6. In Vitro Cytocompatibility Evaluation

**Figure 5:**
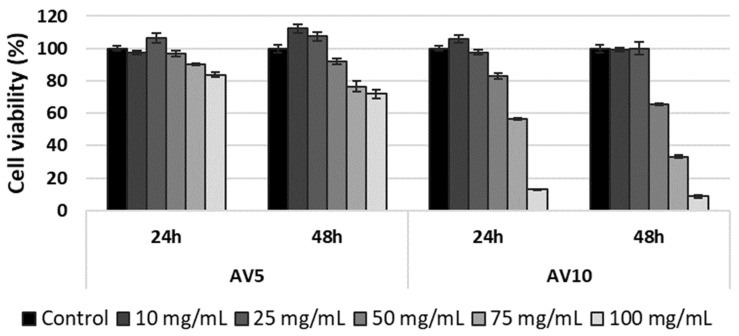
Viability of L929 murine fibroblasts cultivated in the presence of different concentrations of AV5 and AV10, for 24 h and 48 h, evaluated by the MTT assay. Samples were reported to untreated cells (control), considered to have 100% viability. Data were expressed as mean values ± SD (n = 3).

The viability of L929 murine fibroblasts treated with AV5 and AV10 hydrogels was determined using the MTT assay, which measures the activity of mitochondrial dehydrogenases. The results obtained after 24 h and 48 h of cell incubation in the presence of different concentrations of hydrogels ranging between 10 and 100 mg/mL indicated a higher cytotoxic effect of AV10 hydrogel compared to AV5 hydrogel (Figure 5). After 24 h of treatment, AV5 presented a high degree of cytocompatibility at all tested concentrations, the percentages of viability ranging between 106.22% and 83.58%. However, after 48 h, cell viability was maintained above 80% (non-cytotoxic effect) only at concentrations ranging between 10 and 50 mg/mL, whereas a moderate cytotoxicity was observed at higher concentrations, with viability values decreasing to 72.01%. For AV10, a good cytocompatibility was observed only up to the concentration of 50 mg/mL, after 24 h of treatment, and up to 25 mg/mL, after 48 h (Figure 5). At higher concentrations, cell viability significantly decreased below 66% after 24 h (56.47% at 75 mg/mL and 13.06% at 100 mg/mL) and 48 h (65.33% at 50 mg/mL, 33.18% at 75 mg/mL and 8.69% at 100 mg/mL), respectively.

**Figure 6:**
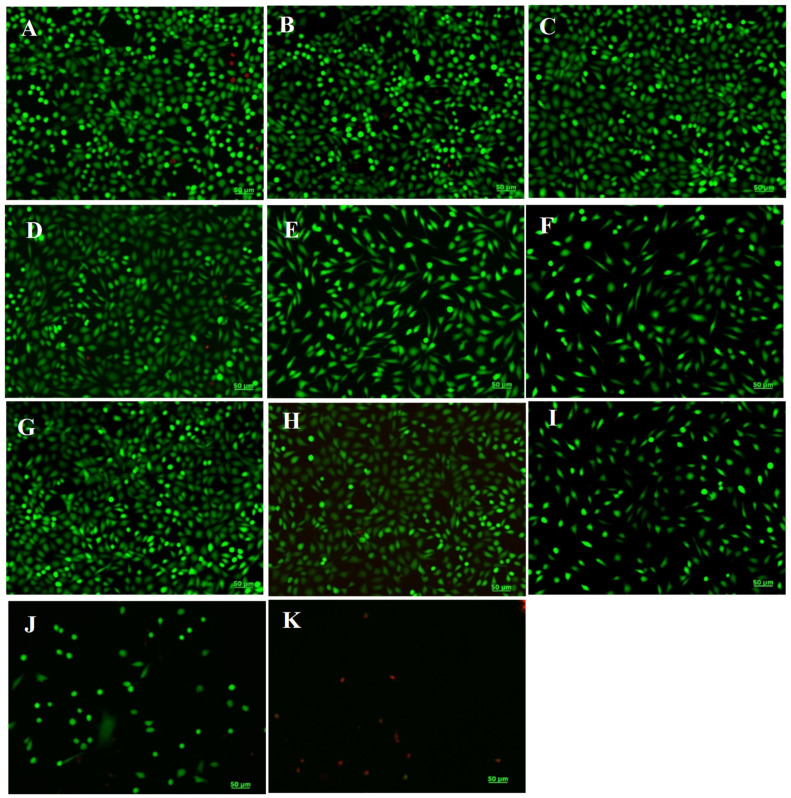
Fluorescent images of L929 live (green) and dead (red) cells, untreated (control, (**A**)) and treated with AV5 (**B**–**F**) and AV10 (**G**–**K**) hydrogels at different concentrations for 48 h. (**B**,**G**)—10 mg/mL; (**C**,**H**)—25 mg/mL; (**D**,**I**)—50 mg/mL; (**E**,**J**)—75 mg/mL; (**F**,**K**)—100 mg/mL. Scale bar = 50 µm.

Fluorescence microscopy was used to evaluate cell morphology and viability after the staining of live cells with calcein (green) and dead cells with ethidium homodimer (red). L929 murine fibroblasts maintained their viability after 48 h of treatment with AV5 hydrogel at concentrations ranging between 10 and 50 mg/mL and with the AV10 sample at concentrations ranging between 10 and 25 mg/mL (Figure 6B–D,G,H). In addition, treated cells maintained their normal phenotype, similar to that of the control, with no significant morphological changes. Cell density was also comparable to that of the control, cells being able to grow and proliferate until reaching an almost confluent monolayer. The proportion of dead cells was very low, suggesting the cytocompatibility of both hydrogels at the above-mentioned concentrations. However, at higher concentrations, the AV5 hydrogel induced a decrease in cell proliferation but with no important morphological changes (Figure 6E,F), whereas the treatment with AV10 induced changes in the morphological appearance of the L929 fibroblasts, with the rounding of most cells or even their massive death (Figure 6I–K).

### 2.7. In Vitro Scratch Assay

**Figure 7:**
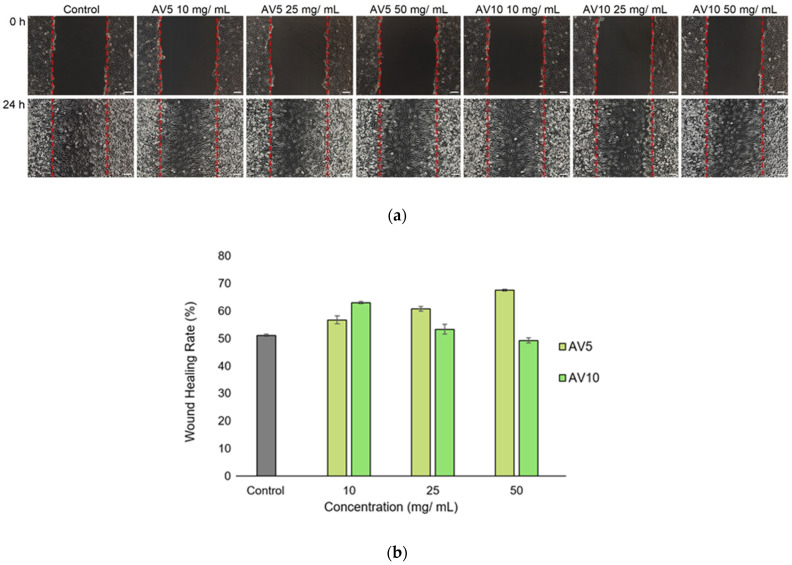
Light microscope images of the L929 cell monolayer (**a**) after in vitro generation of a wound and treatment with different concentrations (10, 25 and 50 mg/mL) of AV5 and AV10 hydrogels, for 24 h. The cell migration in the injured area could be observed. Upper line—t = 0, lower line—after 24 h of treatment. Control was represented by untreated cells. The percentage of wound closure for L929 (**b**) was determined by ImageJ analysis. Data were expressed as mean values ± SD (n = 3).

In vitro scratch assay showed the ability of both AV5 and AV10 hydrogels to accelerate cell proliferation and migration and to induce the closure of a wounded area. This method covers the second phase of the wound healing process, characterized by the proliferation and migration of different types of cells, such as keratinocytes and fibroblasts [69], and is an adequate technique to obtain preliminary results on the wound repair potential of a biomaterial or biocomposite [70,71]. Our results showed that both hydrogels were more effective in repairing the ‘wounded’ L929 monolayer after 24 h of treatment, compared to the control, at all tested concentrations, except for AV10 at 50 mg/mL (Figure 7a). In general, treated cells had a higher ability to migrate and proliferate than untreated ones. Furthermore, the microscopic observations correlated well with the statistical analysis conducted with ImageJ software. Both hydrogels exhibited a higher wound healing rate than that of the control (51.26%), except for AV10 at 50 mg/mL (49.46%) (Figure 7b). In the case of AV5, the wound healing rate increased with increasing concentration (56.95% at 10 mg/mL, 60.92% at 25 mg/mL and 67.75% at 50 mg/mL), whereas for AV10 the wound healing rate decreased with increasing concentration (63.24% at 10 mg/mL, 53.51% at 25 mg/mL and 49.46% at 50 mg/mL). The percentage of wound closure obtained for AV10 at the concentration of 50 mg/mL correlated with the cell viability obtained by MTT assay (65.33%—moderate cytotoxicity), where the treatment did not induce cell death but reduced cell proliferation.

Therefore, the test results suggest that *Aloe vera* based hydrogels are indicated for use as fast-penetrating transdermal dressings through an effective local action of in-tense hydration and for shortening the total healing time.

## 3. Materials and Methods

### 3.1. Materials

*Aloe vera* powder, organic (lyophilized, certified as 100% organic by Ecocert Greenlife according to COSMOS-standard), allantoin powder (purity > 99%, melting point—229 °C), natural BHA salicylic acid (9.8–11.5%, aqueous extract from the bark of the black willow tree) and xanthan gum (raw material), highly pure form (91.0–108.0%) and of high-molecular weight, were purchased from Elemental SRL, Romania, as certified organic products. Deionized water was used as a solvent for hydrogel preparation.

### 3.2. Preparation of the Hydrogels

Two formulations were prepared by dispersing allantoin powder in deionized water, at 80 °C, under vigorous stirring, until dissolution. The solutions were slowly cooled under continuous stirring for 1 h to ambient temperature. Then, under magnetic stirring, salicylic acid and *Aloe vera* powder were gradually added one after the other. After 30 min, xanthan gum was slowly introduced into the solutions as a gelling agent, and the formation of hydrogels occurred almost immediately. Two different samples were prepared with a concentration of 5% and 10% *Aloe vera*, according to Table 3. For future characterization, a part of the hydrogels was poured into Petri dishes and air-dried at room temperature. The prepared samples were denoted as AV5 and AV10, corresponding to the concentration of *Aloe vera* in the samples.

### 3.3. Methods

#### 3.3.1. Determination of Surface pH

To five samples of wet and dry hydrogels from each series, 1 ml of distilled water (pH = 6.5 ± 0.5) was added and kept in contact for 5 min at room temperature. Then, the CONSORT P601 pH-meter electrode (CONSORT^nv^, Turnhout, Belgium) was placed on the surface of the hydrogel formulation. The pH value was displayed, and the results are expressed as mean value ± SD [72,73].

#### 3.3.2. Rheology

At a constant temperature of 25 °C, the viscosity and oscillation behavior of samples AV5 and AV10 were investigated using a Kinexus PRO rheometer equipped with Julabo CF41 cryo-compact circulator. The geometries used were plate-plate type with a diameter of 20 mm and a gap of 0.9 mm. Viscosity curves were obtained at a shear rate ranging from 10^−3^ to 10^3^ s^−1^. Oscillation actions included amplitude shear stress measurements ranging from 0.1 to 100 Pa at a frequency of 1 Hz, as well as frequency sweep measurements at a constant shear stress of 5 Pa. All the rheological data were presented using a logarithmic scale.

#### 3.3.3. Morphology

The morphology of the samples was investigated by scanning electron microscopy (SEM) collecting the secondary electrons signal in a high-resolution field emission Quanta 3D microscope operating in high vacuum mode at an accelerating voltage of 15 kV.

#### 3.3.4. Antimicrobial Activity of Hydrogels

##### The Inhibition Zone Detection

As a first screening method, an adapted Kirby-Bauer assay [74] was carried out on reference strains *S. aureus* (ATCC 25923) and *P. aeruginosa* (ATCC 27853). The bacterial cultures were grown on trypticase soy agar (TSA) nutrient medium and Luria Bertani (LB) nutrient medium by aerobic incubation at 37 °C. The adapted method of agar well diffusion was performed with standardized bacterial suspensions corresponding to 0.5 McFarland density prepared from fresh (24 h) solid cultures of the two bacterial strains. The 2 hydrogel variants, in triplicate, were sterile disposed in the wells obtained with a sterile cork borer. The plates were incubated at 37 °C for 24 h. After the incubation, the diameter of the inhibition zone (mm) was measured, using a classical method (by eye) with a metric transparent ruler [75] with a precision of ±0.5 mm. Each experiment was repeated 3 times. The given value for the diameter of the inhibition zone (mm) is the most frequent value obtained after 3 measurements.

##### The Antibacterial Activity on Planktonic Growth

The antibacterial activity of the *Aloe vera* based hydrogels was evaluated on both Gram-positive, *Staphylococcus aureus* (ATCC 25923) and Gram-negative strains, *Pseudomonas aeruginosa* (ATCC 27853). Samples were UV sterilized. *S. aureus* was grown on trypticase soy agar (TSA) nutrient medium, while *P. aeruginosa* was grown on Luria Bertani agar at 37 °C. The overnight culture was diluted to a final concentration of 1 × 10^8^ colony forming units per mL (CFU/mL) in each well containing AV5 and AV10 hydrogels. After 24 h, the absorbance of the supernatant was assessed at 620 nm to determine the bacterial viability and growth using a Sunrise microplate reader (Tecan).

#### 3.3.5. In Vitro Cytocompatibility Evaluation

In vitro cytocompatibility evaluation of the *Aloe vera* based hydrogels was assessed on L929 murine fibroblasts purchased from the European Collection of Authenticated Cell Cultures (ECACC), using the 3-(4,5-dimethylthiazol-2-yl)-2,5-diphenyltetrazolium bromide (MTT) assay. The extraction medium (100 mg/mL) was prepared by sample incubation in Minimum Essential Medium (MEM) for 24 h at 37 °C and sterile filtration using a 0.22 µm syringe filter. Cells were seeded in MEM medium supplemented with 10% fetal bovine serum (FBS) and 1% antibiotics (penicillin, streptomycin, and neomycin) in 96-well tissue culture plates at a density of 5 × 10^4^ cells/mL and incubated at 37 °C in a humidified atmosphere with 5% CO_2_ for 24 h to allow cell attachment. Subsequently, the culture medium was replaced with different concentrations (10, 25, 50, 75 and 100 mg/mL) of sample extraction medium, and cells were further incubated in standard conditions for 24 h and 48 h, respectively. Then, the cell viability was evaluated by the MTT assay, as previously described by Stefan et al. [71]. Briefly, cells were incubated with 0.25 mg/mL MTT solution (Sigma-Aldrich, Steinheim, Germany) for 3 h at 37 °C. Then, the insoluble formazan crystals were dissolved with isopropanol and the absorbance was measured at 570 nm using the SPECTROstar^®^ Nano microplate reader (BMG, Ortenberg, Germany). The amount of formazan was directly correlated to the number of metabolically active cells. The results were expressed as percentage of viability compared to the negative control (untreated cells) considered 100% viable. Data were presented as the mean of three measurements ± SD.

Cell viability was also examined by fluorescence microscopy using a Live/Dead assay kit (Molecular Probes, Thermo Fisher Scientific, Waltham, MA, USA). Briefly, after 48 h of treatment, cells were washed with PBS and stained with calcein-AM (2 µM) and ethidium homodimer-1 (4 µM), at room temperature for 30 min. Fluorescent images were acquired using an Axio Observer D1 microscope provided with AxioVision 4.6 software (Carl Zeiss, Oberkochen, Germany).

#### 3.3.6. In Vitro Scratch Assay

To evaluate the ability of *Aloe vera* based hydrogels to induce cell migration and proliferation into a wounded monolayer and, therefore, to determine their wound healing effect, L929 murine fibroblasts were seeded in 24-well culture plates at a cell density of 2 × 10^5^ cells/mL and grown to confluence in standard conditions. Then, a linear wound was created in the cell monolayer with a sterile pipette tip and the detached cells were removed by gentle washing with PBS. The extraction medium was added into the wells at different concentrations (10, 25 and 50 mg/mL) and cells were further incubated in standard conditions for 24 h. Light microscope images were obtained at the beginning of the experiment (t = 0) and after 24 h of incubation in order to evaluate the cell migration and the covering of the injured area. Digital images were analyzed using ImageJ 1.51 software in order to quantify the wound healing rate (%).

## 4. Conclusions

In conclusion, the hydrogel based on *Aloe vera* could lead to the adjustment of inflammation, to the intensification of the contraction and to the epithelization of the wound and, finally, to the regeneration of the tissue. The morphological and rheological characteristics, cytocompatibility and cell viability of the two obtained hydrogels were evaluated. SEM images showed a smooth surface, an essential feature to promote better adhesion and interaction of the hydrogel in the wound and surrounding tissues. The rheological profile showed the predominant character of elastic gel. Preliminary results of the scratch wound healing analysis suggest that topical administration of *Aloe vera* based hydrogels may be useful in clinical practice.

Therefore, the test results suggest that *Aloe vera* based hydrogels are indicated for use as fast-penetrating transdermal dressings through an effective local action of intense hydration and for shortening the total healing time. The simple synthesis method used led to obtaining hydrogels with tunable desired properties, with the future possibility of incorporating different drugs or other bioactive compounds into their structure to help and to restore the integrity of damaged tissue.

## Figures and Tables

**Table 1:** pH values of wet and dry samples.

Tested Parameter *	Formulation Code			
	AV5 Wet	AV5 Dry	AV10 Wet	AV10 Dry
pH	5.81 ± 0.03	5.85 ± 0.11	5.93 ± 0.01	5.96 ± 0.03

* Expressed as mean value ± SD.

**Table 2:** The diameter of the zone of inhibition of *S. aureus* and *P. aeruginosa* strains treated with the *Aloe vera* based hydrogels.

	**Diameter of Inhibition Zone (mm)**					
	***S. aureus***				***P. aeruginosa***	
	AV 5	AV 10			AV 5	AV 10
24 h	7	9			5	7

**Table 3:** Composition of transdermal hydrogels.

Samples	AV * (%*w*/*v*)	Allantoin (%*w*/*v*)	Xanthan Gum (%*w*/*v*)	Salicylic Acid (%*v*/*v*)
AV5	5	5	2	1
AV10	10	5	2	1

Deionized water q.s. up to 100%. * Notation: AV = *Aloe vera*.

## Data Availability

Not applicable.
